# Technique for Measuring Power across High Resistive Load of Triboelectric Energy Harvester

**DOI:** 10.3390/mi12070766

**Published:** 2021-06-29

**Authors:** Subhawat Jayasvasti, Panu Thainiramit, Phonexai Yingyong, Don Isarakorn

**Affiliations:** Department of Instrumentation and Control Engineering, School of Engineering, King Mongkut’s Institute of Technology Ladkrabang (KMITL), Bangkok 10520, Thailand; 60601162@kmitl.ac.th (S.J.); panu.th@kmitl.ac.th (P.T.); 62601019@kmitl.ac.th (P.Y.)

**Keywords:** internal resistance, electrical measurement, power measurement, triboelectric energy harvester

## Abstract

This paper proposed a more-accurate-than-conventional measurement technique for determining electrical power across exceptionally high-impedance of triboelectric energy harvester (TEH). The key idea of this proposed technique was to measure the voltage across an introduced, parallelly-connected resistor divider to the oscilloscope instead of the voltage across the harvester. An experiment was set up to verify the measurement accuracy performance of this technique against the ideal theoretical values. The maximum percentage error found was only 2.30%, while the conventional measurement technique could not be used to measure voltage across high impedance TEH at all because the readings were not accurate, i.e., the measurement error would be at least over 10%. Therefore, we concluded that this proposed technique should always be used instead of the conventional measurement technique for power measurement of any TEH. A suggestion that we would like to offer to researchers investigating or developing a TEH is that, in using our measurement technique, a good starting point for a load to probe resistance ratio is 1:10, a ratio that worked well for our TEH test bench that we developed.

## 1. Introduction

Power generation by harvesting waste energy has been investigated intensively in the last decade. Specifically, an energy harvester is required for uses of internet of things (IoTs) applications, including autonomous wireless devices [1,2]. It can reduce the use of conventional battery, whose disposal can create an environmental problem. Its proper recycling process is also expensive. Many kinds of external energy are produced all the time and everywhere (e.g., wind flow, sun light, gradient thermal, and mechanical vibration). Mechanical vibration energy can be captured and converted into electrical power. Research has reported on the mechanical energy harvesting in different transduction principles, e.g., electrostatics, electro-mechanism, and piezoelectricity. Recently, the triboelectric effect has been given much attention because of its potential and advantages as an energy harvester. The working principle of triboelectricity is simply the generation of a static electrical charge by friction or temporary contact based on charge transfer between two materials. However, measuring the electrical power across triboelectric energy harvester (TEH) could be problematic due to its high resistivity.

To be able to determine the maximum electrical power produced by triboelectric energy harvester (TEH), as a power generator, its internal resistance *R_int_* must be known first because the internal resistance *R_int_* dictates the value of a proper resistive output load (i.e., the optimal resistance) to which the generator can deliver. At a proper value of load resistance *R_L_*, an electrical power generator can provide the maximum power. In other words, the maximum power transfer occurs when the load resistance *R_L_* is optimal for the generator.

The optimal value for load resistance can be determined by trying out a range of values for load resistance *R_L_* and picking the one that provides the maximum power transfer or extrapolating to the best value from the value that provided the maximum power transfer in the try-out. Equivalently, we can determine a range of values for the internal resistance *R_int_* from the achieved maximum or near maximum power transfer by varying the values of the load resistance *R_L_* and use a value (exact or extrapolated) in the range to determine the optimal load resistance [3,4,5,6].

However, in practice, power is calculated from voltage reading of an oscilloscope connecting to a TEH, and there is an issue that such voltage readings are inaccurate due to the high impedance of TEH [7,8], which our team had encountered many times in the past. Therefore, we designed and developed a technique for accurate oscilloscope voltage measurement of high-impedance TEH and all necessary mathematical equations for calculating power of TEH under this technique. Later in this section, we explain this problem in detail in Section 1.1, and our rationale behind the design of this technique in Section 1.2.

### 1.1. High Impedance of Triboelectric Energy Harvester and Its Corresponding Inaccurate Power Measurement Problem

Internal resistance of a triboelectric generator *R_int_* is a specific characteristic of the generator, directly related to the amount of current *i* that flows through it. An internal resistance of a triboelectric generator *R_int_* is typically very high, in the order of 10 MΩ and over [8,9,10,11,12]. In principle, a load resistance *R_L_* that matches the internal resistance *R_int_* of a device allows maximum transfer of power, to the load and is considered the optimal resistance for the generator.

Typically, a triboelectric energy harvester has a high impedance compared to the impedance of a voltage measurement device, as shown in Table 1 with the reference papers.

**Figure 1:**
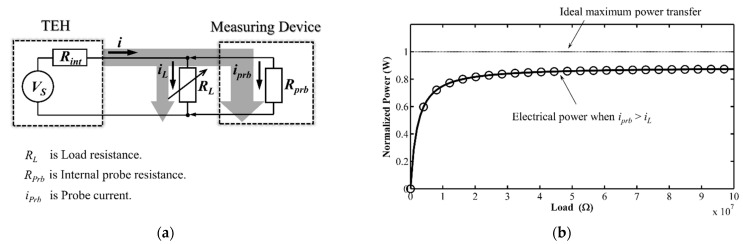
(**a**) Schematic diagram of the conventional measuring technique and (**b**) plot of maximum power versus load resistance under this technique.

Due to this high impedance, measuring the voltage of a harvester by conventionally connecting it across the probes of a measuring device would not give a stable, accurate reading [7]. This problem is illustrated in Figure 1, which also clearly shows the extent of deviation from the theoretical maximum power limit.

### 1.2. The Rationale behind Our Developed Measurement Scheme 

The most common voltage measurement device for an energy harvester is an oscilloscope. In principle, an oscilloscope must not shunt any significant current through itself, i.e., its probe resistance *R_prb_*. However, since the internal resistance of an energy harvester, *R_int_*, is considerable higher than a typical *R_prb,_* a significantly larger amount of current will flow through the probes than it is supposed to, causing erroneous voltage readings across the load resistance *R_L_*, and hence erroneous calculated power. We thought that we could reduce that flow through the probes by shunting some of it to a parallelly connected resistor divider and then measure the voltage across that resistor divider instead of across the TEH itself. Next section shows the derivation of all mathematical equations involved in the calculation of voltage, current, and power in this measurement scheme from Kirchhoff law.

## 2. Kirchhoff Voltage Law and Derivation of Mathematical Relationships among Measurement Variables

**Figure 2:**
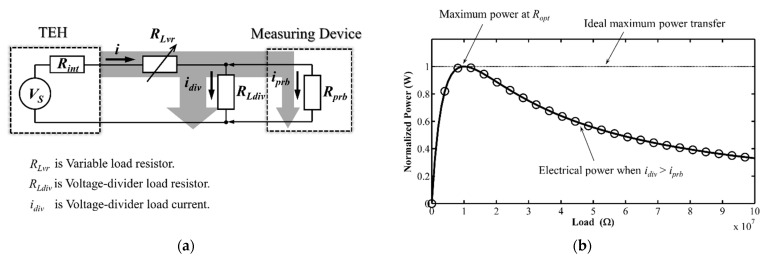
(**a**) Schematic diagram of the developed technique and (**b**) plot of maximum power versus load resistance under the developed technique.

The developed technique was designed to address the measurement problem when an oscilloscope performs too much non-ideally as an open circuit measuring device. Hence, the theoretically predicted electrical metrics of the configuration (see Figure 2a) of our technique for measuring the voltage across a parallelly-introduced resistor divider could be derived from Kirchhoff voltage law [17]. The following paragraphs show the derivation of every mathematical expression that we used to calculate the predicted values.

Let *P_L_* be the electrical power across the load resistance and *R_L_* be the load resistance. Their dependence on each other is expressed by Equation (1). 

The load current value is calculated from the voltage generated by TEH across the load resistance (*R_int_, R_Lvr_, R_Ldiv_,* and *R_prb_*), which is based on Kirchhoff’s law, as shown in Equation (2). The key voltage-divider resistor *R_Ldiv_* value that we used in our technique is taken into account in Equation (2), based on the application of Kirchhoff’s law to the configuration of our technique,

(1) $$P_{L}=i^{2}\times R_{L},$$

(2) $$P_{L}={\left(\frac{V_{S}}{R_{int}+(R_{Lvr}+(R_{Ldiv}//R_{prb}))}\right)}^{2}\times \left(R_{Lvr}+(R_{Ldiv}//R_{prb})\right).$$

In this developed technique, the actual voltage measured by the oscilloscope was the voltage across the divider resistor, Vread. To calculate the theoretical voltage generated by the TEH across the load resistor, Vout, Equation (3) was derived from Kirchhoff law in the same way explained above,

(3) $$V_{out}=\frac{V_{read}\times R_{L}}{\left(R_{Ldiv}//R_{prb}\right)},$$

(4) $$V_{out}=\frac{V_{read}\times \left(R_{Lvr}+(R_{Ldiv}//R_{prb})\right)}{\left(R_{Ldiv}//R_{prb}\right)}.$$

as well as substituting this TEH voltage value, *V_out_*, into Equation (5) and then calculating out the electrical output power generated by the TEH generator from the Equation (6),

(5) $$P_{L}=\frac{V_{out}^{2}}{R_{L}},$$

(6) $$P_{L}=\frac{V_{out}^{2}}{\left(R_{Lvr}+(R_{Ldiv}//R_{prb})\right)}.$$

In addition, this developed technique can also be used to calculate the internal resistance of the energy harvester (Equation (8), derived from voltage divider Equation (7)) from the open circuit voltage and the voltage across the load (R_*Ldiv*_//R_*prb*_).

(7) $$V_{read}=\frac{V_{S}\times (R_{Ldiv}//R_{prb})}{R_{\mathrm{int}}+(R_{Ldiv}//R_{prb})},$$

(8) $$R_{\mathrm{int}}=\frac{\left(V_{S}-V_{out})(R_{Ldiv}//R_{prb}\right)}{V_{out}}.$$

## 3. Materials and Methods

### 3.1. Materials

This section describes the details of the materials used in the validation experiment, listed in Table 2 below. 

### 3.2. Methods

This section describes the experiment that verified the accuracy of the developed technique and the experimental setup. 

This experiment was to reliably measure voltage across the load connected to oscilloscope probes, for verifying the validity of our suggested 1:10 ratio of R_*Ldiv*_:R_*prb*_ (explained in the Section 4.) for an adequate match between such kinds of resistors. 

**Figure 3:**
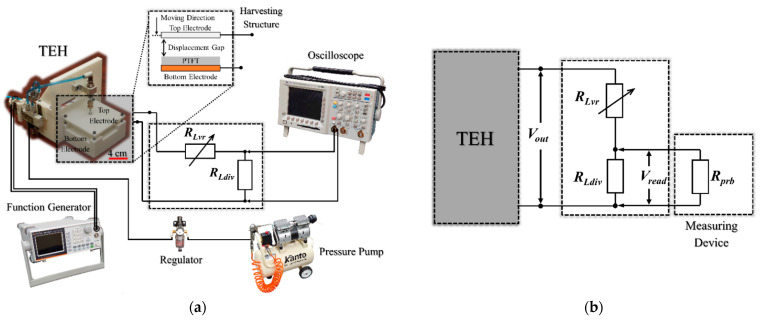
Schematic diagram of (**a**) experimental set-up and (**b**) circuit schematic of measurement technique.

**Figure 4:**
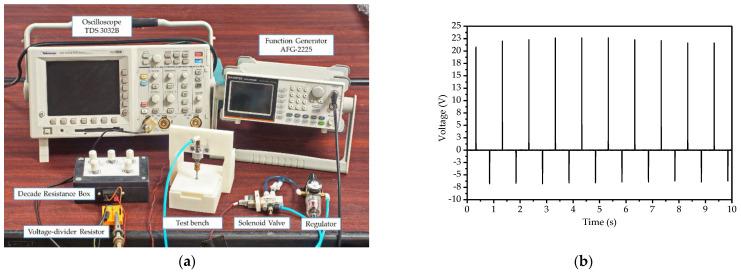
(**a**) Experimental setup test bench; (**b**) voltage signal measured from the developed technique.

The experimental test bench and all components were constructed following exactly the procedures and specifications shown in schematic diagram in Figure 3 (reported in a previous paper by Thainiramit et al. [18]). The pre-set parameter values are listed in Table 2. The experimental setup test bench and the typical voltage signal measured from the developed technique are shown in Figure 4.

**Figure 5:**
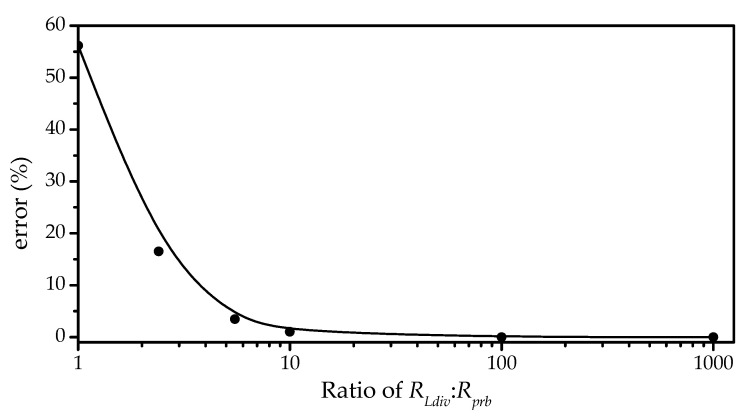
Plot of measurement errors of output power, *P_L_*, versus six different ratios (1:1, 1:2, 1:5, 1:10, 1:100, and 1:1000) of voltage-divider resistance *R_Ldiv_* to probe resistance *R_prb_*.

The measurement error could be calculated from a mathematical expression of an ideal power measurement, *P_Li_*, minus the power measured by the developed technique across the load, *P_Lm_* in Equation (9). In this study, the ideal power measurement excluded the probe resistance, i.e., *R_prb_* was set to infinity. When using the parameter according to Table 2 would get the plot as shown in Figure 5.

(9) $$\%error=\left|\frac{P_{Li}-P_{Lm}}{P_{Li}}\right|\times 100\%$$

(10) $$\%error=\left|1-\frac{R_{Lvr}+R_{Ldiv}}{R_{Lvr}+(R_{Ldiv}//R_{prb})}\right|\times 100\%$$

## 4. Results and Discussion

**Figure 6:**
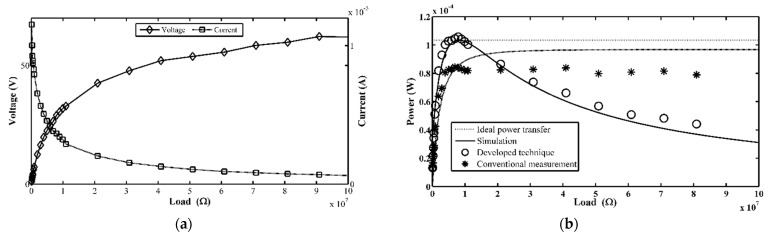
(**a**) Experimental results of output voltage and current; (**b**) plots of experimental results of electrical power obtained from the developed technique, results obtained from the conventional measurement technique, and ideal measurement.

This section mainly discusses the main results of the experiment detailed in the previous section. The experimental results demonstrate that the developed technique was more accurate, i.e., with smaller error, in determining the TEH output power than the conventional technique, as illustrated in Figure 6 (exact numerical values of all parameters are tabulated in Table 3). Moreover, it can be seen clearly in Figure 6b that the curve of measured power versus load resistance from this proposed technique approaches closer to ideal measurement curve than the curve of power measured by the conventional technique. Therefore, we concluded that the experimental results successfully verified that the developed technique was better suited for measuring output power of any high impedance energy harvester than the conventional technique. 

Finally, from our preliminary investigation into finding a proper ratio between the voltage-divider resistor *R_Ldiv_* to the probe resistance *R_prb_* for the developed technique, we obtained 1:10 as a good value for the technique. Although a lower ratio may reduce the current flowing through the probe, it will also reduce the voltage across the resistor divider in the configuration of our technique, hence the inherent noise of the oscilloscope will limit its usefulness, i.e., it makes the technique less sensitive. Therefore, we suggest new researchers to use the proposed technique instead of the conventional technique for measuring output power of high impedance energy harvester in their study as well as selecting a good ratio between the voltage-divider resistor *R_Ldiv_* to the probe resistance *R_prb_* by starting with an initial ratio of 1:10 for such resistors. A lower ratio may or may not provide more accurate readings. The new researchers should check whether a lower ratio may give more accurate and stable readings, but this initial ratio was satisfactory for our technique and setup.

## 5. Conclusions

This study was an experimental study to verify the low percentage measurement error offered by a proposed technique for measuring power generated by high impedance triboelectric generator. The technique was to measure the voltage across an introduced, parallelly-connected resistor divider to the oscilloscope instead of the voltage across the TEH devices. The experimental results confirm that the proposed technique truly provides more accurate power determination than the conventional technique. The 1:10 voltage-divider resistor *R_Ldiv_* to the probe resistance *R_prb_* ratio is the proper value for the practical measurement. However, it should be noted that higher resistances are more sensitive to noise. Findings from this work may directly benefit new developers in the field of triboelectric generator in their effort to accurately measure the electrical characteristics of their TEH devices.

## Figures and Tables

**Table 1:** Internal resistance of several existing triboelectric energy harvesters.

Reference	Voltage	Power	Internal Resistance
Shamsuddin et al., 2019 [13]	40.0 V	17.0 µW	60 MΩ
Mule et al., 2019 [14]	774.6 V	10.0 mW	60 MΩ
Jurado et al., 2017 [15]	3.8 V	307.8 µW	10 MΩ
Kim et al., 2017 [16]	13.0 V	201.0 µW	20 MΩ
Yang et al., 2016 [8]	749.4 V	9.4 mW	60 MΩ

**Table 2:** Details of materials and mechanical parameters used in the experiment.

Material	Detail
Polytetrafluoroethylene (PTFE) dimension ^1^	50 × 50 × 1 mm^3^
Displacement gap	4 mm
Top electrode material	Aluminum foil (3M 425)
Top electrode dimension ^1^	50 × 50 × 0.12 mm^3^
Bottom electrode material	Copper foil (MT 8113C)
Bottom electrode dimension ^1^	50 × 50 × 0.1 mm^3^
Voltage-divider resistor, *R_Ldiv_*	1 MΩ
Equivalent voltage source, VS ^2^	61 V
Optimal resistance	8.9 MΩ
Probe’s internal resistance, *R_prb_*	10 MΩ
**Mechanical parameter**	
Regulated air pressure	600 kPa
Direct mechanical force	17.2 N
Spring constant	263 N/m
Input frequency	2 Hz
Initial acceleration ^3^	0.93 g
Final acceleration ^3^	4.28 g

^1^ Width × length × thickness. ^2^ Using Thévenin’s theorem. ^3^ 1 *g* implies 9.81 m/s^2^.

**Table 3:** Theoretical and measured maximum power and load resistance at maximum power.

Item	Theoretical	Measured	Percentage Error
Maximum power transfer	103.36 µW	105.74 µW	2.30
Resistance at maximum power	8.108 MΩ	8.90 MΩ	9.77

## Data Availability

Not applicable.
